# Adolescents’ lived experiences of facility-based childbirth in rural northern Uganda: A qualitative study

**DOI:** 10.1177/17455057261468279

**Published:** 2026-07-09

**Authors:** Samson Udho, Sheila Elizabeth Clow

**Affiliations:** 1 Division of Nursing and Midwifery, Department of Health and Rehabilitation Sciences, Faculty of Health Sciences, 63726 University of Cape Town, South Africa; 2 Department of Midwifery, Faculty of Nursing and Midwifery, Lira University, Lira, Uganda

**Keywords:** adolescents, childbirth experiences, facility-based care, respectful maternity care, Uganda

## Abstract

**Background:**

Adolescents in rural northern Uganda face unique challenges during facility-based childbirth, yet their experiences remain underexplored.

**Objective:**

To explore adolescents’ experiences during facility-based childbirth in rural northern Uganda.

**Design:**

A qualitative exploratory descriptive study.

**Methods:**

In-depth interviews were conducted with 14 purposively sampled adolescents aged 15–19 years who gave birth in public health facilities in rural northern Uganda. Data was collected using a pre-tested interview guide with open-ended questions and analysed inductively using thematic analysis and reported according to the COnsolidated criteria for REporting Qualitative research (COREQ) guidelines.

**Results:**

Participants’ age ranged from 15 to 19 years and they were predominantly primiparous (78.6%). Their experiences were mixed, encompassing both positive and negative aspects. Negative experiences fell under two themes: (1) disrespect and abuse (physical and verbal abuse, neglect, non-consented care, disempowerment, and bribery) and (2) health facility constraints (dirty toilets, unstable water supply, and shortages of medicines and supplies). Positive experiences were categorized into four themes, (1) effective communication (clear interactions and information sharing), (2) dignity and respect (respectful and friendly care), (3) supportive care (hospitality, encouragement, companionship, and timely care), and (4) facility hygiene (clean labor suites and wards despite some unhygienic toilets).

**Conclusions:**

Adolescents’ facility-based childbirth experiences in rural northern Uganda were mixed, marked by both respectful care and mistreatment. While some received supportive and dignified care, others faced abuse, neglect, and facility constraints. Strengthening respectful maternity care and improving facility conditions are essential for enhancing adolescent birth experiences.

## Introduction

Adolescence is a critical period of transition marked by physical, emotional, and social changes that influence reproductive health outcomes.^1,2^ Globally, adolescent pregnancy remains a significant public health concern, with low- and middle-income countries (LMICs) bearing the highest burden.^3–5^ According to the World Health Organization (WHO), every year an estimated 21 million girls aged 15–19 years in developing regions become pregnant and approximately 12 million of them give birth, with sub-Saharan Africa accounting for nearly half of these pregnancies and births.^4,5^ In Uganda, adolescent pregnancy rates stand at 25%, with rural areas experiencing disproportionately high prevalence due to socio-economic vulnerabilities, cultural norms, and limited access to sexual and reproductive health services.^6–8^

Facility-based childbirth is widely recognized as a key strategy for improving maternal and neonatal health outcomes.^9,10^ However, evidence suggests that adolescents face unique challenges when accessing and utilizing facility-based maternity services.^10,11^ Documented barriers such as mistreatment, inadequate support, and poor health facility conditions,^10,12–17^ may deter adolescents from seeking skilled birth attendance, and negatively impact their childbirth experiences.^13,18^

Despite global and national commitments to promoting respectful maternity care (RMC),^19–21^ adolescents in rural settings remain vulnerable to mistreatment during childbirth. Research on facility-based childbirth experiences among adolescents in Uganda, particularly in rural northern regions, remains limited. Rural northern Uganda was selected as the study setting due to its high adolescent pregnancy rates (41.2%-62.4%),^22,23^ limited access to quality maternal healthcare services,^24,25^ and the socio-cultural factors that influence childbirth experiences in this region.^26–28^ The diversity of cadre offering maternity care as well the proportion of male and female midwives (40% versus 60% respectively) trained to offer maternity care also influence the choice of study context.^29,30^ Understanding adolescents’ experiences is essential for informing policies and interventions aimed at improving maternal care for this vulnerable group.

This study explored adolescents’ experiences during facility-based childbirth in rural northern Uganda, highlighting both positive and negative aspects of care. Findings provided insights into the nature of support and mistreatment experienced, facility constraints, and the factors influencing adolescents’ perceptions of care. By identifying areas for improvement, the study contributed to ongoing efforts to enhance respectful maternity care and improve maternal health outcomes among adolescents in Uganda and similar settings.

## Materials and methods

### Study design and setting

A qualitative descriptive research (QDR) design^31,32^ was employed to explore adolescents’ experiences of care during childbirth in public health facilities in Lira District, northern Uganda, from March to October 2023. The findings are reported according to the COnsolidated criteria for REporting Qualitative research (COREQ)^33^ (Appendix 1). QDR is an exploratory approach aimed at describing and summarizing specific events experienced by individuals or groups in everyday language. This design is influenced by the phenomenology of Edmund Husserl,^34^ particularly the concept of bracketing.^31,32^ Given that the researchers are midwife educators with pre-existing assumptions about women’s childbirth experiences, bracketing was a crucial step to minimize potential bias. This study was part of a mixed-method study that examined adolescents’ perceptions of person-centred maternity care during facility-based childbirth in rural northern Uganda and how their perceptions influenced their satisfaction with care and future childbearing intentions and explored the experiences and drivers of adolescents’ experiences of care during childbirth.

The study took place in Lira District, located 342 kilometers (212 miles) north of Kampala, the capital of Uganda. Covering a total area of 1,326 square kilometers, Lira District includes 1,286 square kilometers of land, with the remainder consisting of water and swamps.^35^ The district is divided into two counties: Erute North and Erute South, separated by Lira City (^36^. Erute North includes the sub-counties of Aromo, Agweng, and Ogur, while Erute South consists of Barr, Agali, and Amach sub-counties. Each of these sub-counties has either level III or IV health facilities where the study participants gave birth.^36^ Level III health facilities offer antenatal, intrapartum, and postnatal care services. It also offers laboratory services and Basic Emergency Obstetric and Newborn Care (BEmONC).^37^ Level IV health facilities offer antenatal, intrapartum, and postnatal care services. It also offers laboratory services and Comprehensive Emergency Obstetric and Newborn Care (CEmONC).^37^

### Study participants and sample size estimation

The study focused on female adolescents aged 14 to 19 years who had given birth in the previous two to six weeks at public health facilities in Lira District offering maternity services and who could communicate in either Lango (the local dialect) or English. Participants who were ill, not at home or busy with their other activities during the data collection period were excluded. The study also excluded those who were staff members in the selected health facilities; a first-degree relative (mother, sister, cousin) to a staff member in the selected health facilities; and those with a probable diagnosis of postnatal depression at the time of data collection based on a three-point Edinburgh Postnatal Depression Scale.^38^

Using purposive sampling, 14 participants were selected based on their ability to address the research aims and their knowledge and experience regarding the phenomenon under investigation.^39^ The sample size was determined using the principle of information power, which posits that a sample with a greater amount of relevant information can yield sufficient insights with fewer participants.^40^ Consequently, a final sample size of 14 was deemed adequate upon data saturation. The homogeneity of the study population and the use of rich, in-depth interviews supported early saturation, consistent with phenomenological research standards.

## Participants’ recruitment

Eligible study participants were identified from the postnatal clinic or maternity ward of the health facilities with the support of facility staff members. The research assistant collected the home addresses and mobile phone numbers of both the prospective study participants and their next of kin. The interviewer then conducted a follow-up between the second and sixth week postpartum at participants’ homes. During this time, written informed consent and or assent was obtained after explaining the purpose, benefits, and risks of participating in the study and subsequently recruited in the study. A total of 17 prospective participants were recruited for the study although only 14 were finally interviewed.

### Data collection

After ethical and administrative approvals, data were collected through face-to-face in-depth interviews (IDI) guided by a pre-tested semi-structured framework that included open-ended questions (Appendix 2). This approach enabled the researcher to record both verbal and nonverbal cues relevant to adolescents’ experiences during childbirth. Additionally, it allowed the researcher to probe participants for a more nuanced understanding of the significance of their experiences.

The data were collected by SU (male), a nurse-midwife by profession working as a midwife educator at a public university in Uganda, who has five years of experience in conducting qualitative research. He has received training in responsible research conduct, protection of human subjects, and conducting in-depth interviews. Although the interviewer had no prior relationship with any of the participants, he established rapport during the data collection process. All interviews were conducted in the Lango language, as preferred by the participants. With their permission, the interviews were audio-recorded using a smartphone. The interviews took place in participants’ homes and lasted between 45 and 90 minutes. Field notes were taken to record participants’ body language, mood, and behaviours during the interviews. At the end of each interview, the interviewer summarized the discussion and asked participants to confirm whether it accurately reflected their experiences.

### Data management and analysis

Audio-taped interviews were reviewed at the conclusion of each in-depth interview (IDI) to ensure that the captured information aligned with the study objectives. The recorded IDIs were transcribed *verbatim* in the *Lango* language and subsequently translated into English. To verify the accuracy of the translations, 50% of the English transcripts were back translated into *Lango* by a different translator. The translations were found to be accurate and reliable, making further back-translation of the remaining interviews unnecessary. The transcripts were then imported into Atlas Ti version 7.5, a qualitative data management software, for analysis.^41^ Data analysis was conducted using the six steps of reflexive thematic analysis technique outlined by Braun and Clarke.^42^ The six steps of reflexive thematic analysis by Braun and Clarke include: (1) familiarization with the data, (2) generating initial codes, (3) searching for themes, (4) reviewing themes, (5) defining and naming themes, and (6) producing the report.^42^ Semantic data coding was performed independently by two coders, following the guidelines for inductive thematic analysis.^42^ Themes were developed based on the frequency of codes and the adequacy of data extracts to support them. Any discrepancies in codes and themes were resolved through discussions among the authors. The data were presented as direct quotes, accompanied by the context from which they were extracted.

### Trustworthiness

Rigor and trustworthiness in this study were maintained through multiple strategies aligned with qualitative research standards.^43,44^ Credibility was enhanced through member checking by restating or summarizing participants’ responses during the interview for clarification or confirmation, and maintaining an audit trail. Transferability was ensured by selecting diverse participants capable of rich expression and providing thick descriptions of the study context and methods. Dependability was supported by using audio recordings and transcripts, with continuous supervision and auditing by the researcher’s supervisor. Confirmability was achieved through reflexivity, participant debriefing, and audit trails using Atlas Ti software. Reflexivity was ensured through critical self-appraisal, journaling, and regular review of audio data to minimize bias and ensure findings reflected participants’ authentic experiences.

### Ethics approval and consent to participate

Ethical approval for this study was obtained from the Human Research Ethics Committee of the University of Cape Town Faculty of Health Sciences (HREC REF: 310/2022) and the Gulu University Research and Ethics Committee, Uganda (GUREC-2022-480). Additional authorization was granted by the Uganda National Council of Science and Technology (HS2727ES), as well as relevant district and health facility authorities. Written informed consent was obtained from participants aged 18–19 years. For participants younger than 18 years, written parental or guardian consent and participant assent were secured. Confidentiality was ensured through the use of pseudonyms and secure, password-protected data storage. Participants were informed of their right to withdraw at any time without consequences. All data collection procedures adhered to COVID-19 prevention guidelines.

## Results

Seventeen prospective participants were initially sampled for the study. One participant declined to provide consent, and two were lost to follow-up. Data saturation was achieved during the 14^th^ interview, after which data collection was stopped.

### Characteristics of study participants (N=14)

The average age of the study participants was 18.7 years, most of whom had attained primary education (92.86%) and had given birth once (78.57%). Half of the participants (50%) gave birth (spontaneous vaginal birth (SVB) in a level III health centre (H/C III) while the other half gave birth in a level IV health facility (H/C IV) (Table 1).

**Table 1.** Characteristics of study participants (N=14).

Pseudonyms	Age	Level of education	Marital status	Occupation	Parity	Facility H/C level	Mode of birth
Emily	17	Primary	Cohabiting	Farmer	02	H/C III	SVB
Chloe	19	Primary	Married	Farmer	01	H/C III	SVB
Grace	18	Primary	Cohabiting	Farmer	01	H/C III	SVB
Lily	17	Primary	Cohabiting	Housewife	01	H/C III	SVB
Sophia	17	Primary	Married	Farmer	01	H/C III	SVB
Harper	19	Primary	Cohabiting	Housewife	01	H/C IV	SVB
Mia	18	Primary	Married	Housewife	01	H/C IV	SVB
Isabella	15	Primary	Cohabiting	Housewife	01	H/C IV	SVB
Taylor	17	Primary	Cohabiting	Tailor	01	H/C IV	SVB
Campbell	19	Primary	Cohabiting	Farmer	02	H/C III	SVB
Amelia	18	No education	Separated	Farmer	02	H/C IV	SVB
Ella	16	Primary	Cohabiting	Farmer	01	H/C III	SVB
Scarlett	18	Primary	Cohabiting	Farmer	01	H/C IV	SVB
Susan	18	Primary	Cohabiting	Farmer	01	H/C IV	SVB

### Adolescents’ lived experiences of facility-based childbirth

**Figure 1.**
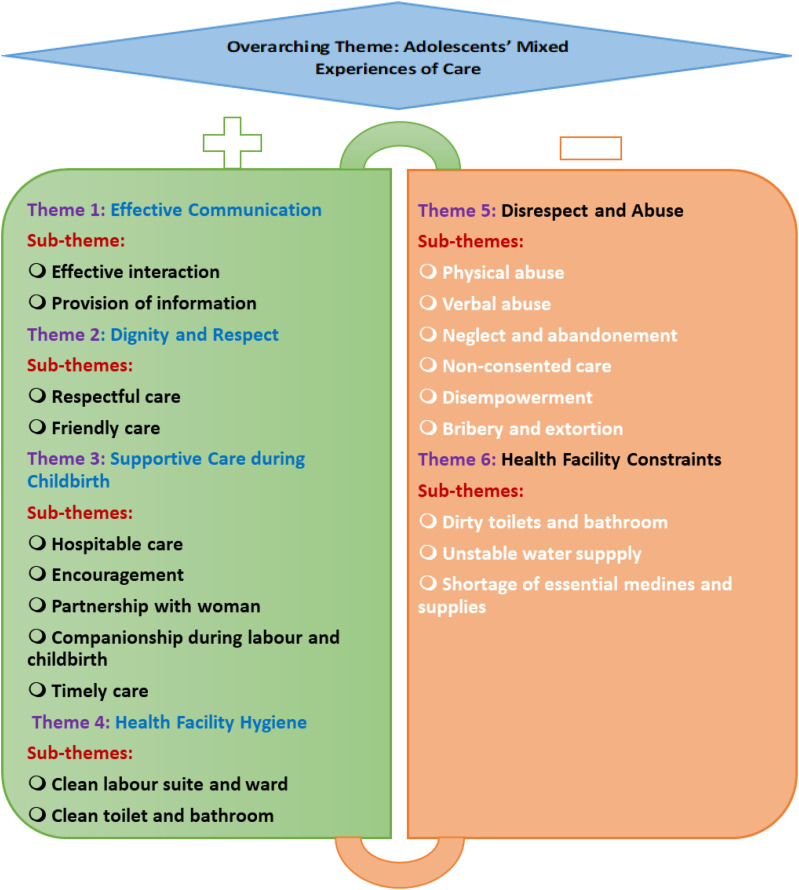
Thematic map of experiences of care during childbirth among study participants.

Mixed experiences of care were identified as the overarching theme. This comprised four positive themes and two negative themes as summarised in Figure 1 with details of the thematic map in Appendix 3.

The themes relating to the positive experiences are presented below followed by the themes relating to the negative experiences.

### Positive experiences of facility-based childbirth

#### Theme 1: Effective communication

Effective communication during childbirth plays a crucial role in shaping the overall birthing experience for adolescents. Adolescents expressed that the effective communication they had with the care providers and the information that they provided during labour and childbirth assisted them to enjoy their birthing experience.

##### Sub-theme 1.1: Effective interaction

Most of the participants interviewed expressed positive experiences of care related to cordial interactions with healthcare providers during their childbirth journey. They highlighted that the midwives talked to them in a friendly manner and communicated respectfully as typified by responses from these participants:

*“She was humble while talking to me and was not rude. They were talking calmly to me.”* (Scarlett, 18-year-old, Para 1)

“*We were even interacting well with joy and I would see and tell those people were liking me because our conversation was going on well*.” (Ella, 16-year-old, Para 1)

The positive interactions with adolescents resulted in words of encouragement as in the case of a teenager giving birth for the second time:

*“She was interacting well with me and she said I should persevere and I will give birth well.”* (Amelia, 18-year-old, Para 2)

How the healthcare provider interacted with the adolescents influenced their experience of care during the process of labour and childbirth. Most of the adolescents did not have any problem with how the midwives communicated with them as noted below:

*“She told me nothing that would make me have problems with her.”* (Chloe, 19-year-old, Para 1)

##### Sub-theme 1.2: Provision of information

The responsibility of answering questions and addressing concerns usually falls on the skilled health personnel who look after the labouring woman. Midwives are suited for this role since they spend the most time with women in labour. Providing health information is critical in creating a positive birth experience, especially for adolescents who might be giving birth for the first time.

*“They kept telling me even the time I was giving birth they told me that I should push the baby [when I feel the contraction is strong].”* (Amelia, 18-year-old, Para 2)

The recurring use of the phrase “they kept telling me” underscores a continuous and supportive communication flow. For some adolescents, telling them when they would give birth, and the progress of labour greatly improved their experiences of childbirth:

*“I was told there was progress [participant smiled] and I was asked to be walking around. I then walked around but reaching around 7:00 pm, things became hot and I gave birth at 9:00 pm.”* (Grace, 18-year-old, Para 1)

*“I was told there is some progress in the labour process and then the baby started pushing out and I continued to also push but I was told my pelvis is narrow [and she was given an episiotomy].”* (Isabella, 15-year-old, Para 1)

#### Theme 2: Dignity and respect

The theme of dignity and respect had two distinct yet interwoven sub-themes of respectful care and friendly care.

##### Sub-theme 2.1: Respectful care

Participants emphasised the nuanced ways in which respect was demonstrated, highlighting a holistic approach to care that extended beyond verbal communication. This was embodied by a quote from one of the participants who was an orphan and came to give birth in the hospital alone:

*“When I reached, the midwife gave me the bed where I was admitted. She [midwife] then asked me to pick the urine mat [mackintosh] and go with it to labour suite and she examined me very well. She [midwife] was humble while talking to me”* (Scarlett, 18-year-old, Para 1)

One of the youngest participants, Ella aged 16 years perceived and reported respect while receiving care as noted below:

*“…there was maximum respect”* (Ella, 16-year-old, Para 1)

While participants were not sure how they would be treated during childbirth, they reported respectful care with a lack of mistreatment as illustrated by quotes from some of these participants:

*“I was not slapped at all. There was no issue and the nurse was the one who helped me to push the baby without any problem, they didn’t quarrel with me, and I wasn’t told anything. I gave birth smoothly because I didn’t go through hardship it was just okay”.* (Sophia, 17-year-old, Para 1)

*“When I was in bed they never shouted at me, if the baby tries to come out and again goes back, they never slapped me.”* (Scarlett, 18-year-old, Para 1)

*“They didn’t shout at or do something bad [she looked timid throughout the interview].”* (Isabella, 15-year-old, Para 1)

##### Sub-theme 2.2: Friendly care

Many participants indicated a positive shift in the caregiver’s approach, signalling a transition from professional care to a more personal, friendly connection. The adolescents experienced friendly care which was perceived in the form of general love and liking for them during care. This suggested a nurturing environment, where emotional well-being was recognised as integral to overall health as reported by some of these participants:

**“***She started being friendly to me during my pregnancy…. Ever since I started my antenatal visit, she showed me love.”* (Chloe, 19-year-old, Para 1)

*“She loved me at a higher level and then told me to deliver well and then I delivered well without complications.”* (Sophia, 17-year-old, Para 1)

*“I would see and tell those people were liking me. They showed me love.”* (Ella, 16-year-old, Para 1)

#### Theme 3: Supportive care during childbirth

Adolescent mothers received appropriate support during childbirth which was in the form of hospitable care, encouragement, partnership, companionship, and timely care.

##### Sub-theme 3.1: Hospitable care

Most of the participants expressed positive experiences related to the hospitality they received during childbirth. The hospitable care was seen by adolescents as a warm welcome and respectful reception upon arrival at the health facility:

“Labour pain started on Monday night and then I went to the hospital on Tuesday around 4:30 pm, we were well received, and I was given necessary services and I never got any problem.” (Susan, 18-year-old, Para 1)

*“When I reached the nurse welcomed me and treated me very well up to the time that I gave birth* ***.”*** (Chloe, 19-year-old, Para 1)

*“I reached the hospital and the nurse [midwife] who received me was in the rank of a sister [senior midwife]. She received me so well* ***”*** (Sophia, 17-year-old, Para 1)

##### Sub-theme 3.2: Encouragement

The healthcare providers encouraged and reassured the adolescents during labour which fostered a positive experience during childbirth. Some participants were reassured about the outcome of pregnancy. This encouraged them to go through the experience of childbirth as teenage mothers as cited by these participants:

*“She said I should persevere [the labour pain] and that I will give birth well [implying there will be no complication].”* (Amelia, 18-year-old, Para 2)

*“If the baby pushed, she would say, you persevere”.* (Grace, 18-year-old, Para 1)

For some adolescents, the absence of complications during labour was used by skilled health personnel to motivate them. Some healthcare providers used the fact that in recent times, adolescents were having normal spontaneous vaginal birth even more than older women to inspire adolescents in labour.

*“The nurse told me that my childbirth would be okay without any complication and also said that young girls are giving birth without complications compared to old women”* (Sophia, 17-year-old, Para 1)

Some participants expressed gratitude to the skilled health personnel for encouraging them to ambulate throughout labour which resulted in a positive birth experience:

*“She also encouraged me to exercise by helping me to walk around while talking to me happily.”* (Chloe, 19-years-old, Para 1)

##### Sub-theme 3.3: Partnership with the adolescents

Participants emphasised the importance of skilled health personnel forming a partnership with them during the childbirth process. This collaboration in the form of physical presence and quality of care, contributed to a sense of trust and support as typified by feedback from some of the participants:

*“One important thing that I know is the nurse [midwife] was on my side. She gave me hope and confidence to give birth without any problem.”* (Sophia, 17-year-old, Para 1)

*“When I entered the labour suite, I immediately climbed onto the bed where both midwives were waiting for me. And then, without any delay, I gave birth”* (Harper,19-year-old, Para 1)

*“The nurse was there [during labour and childbirth] and she waited to receive the baby.”* (Ella, 16-year-old, Para 1)

Some of the midwives cleaned up the mothers and supported them so that they did not fall off the bed while wrestling with labour pain on the delivery bed:

*“The midwife was with me up to the time I gave birth. She even cleaned me up.”* (Scarlett, 18-year-old, Para 1)

*“At some point when I would feel like falling down the nurse would support me.”* (Chloe, 19-year-old, Para 1)

##### Sub-theme 3.4: Companionship during labour and childbirth

Besides the partnership the adolescents had with the midwives during childbirth, most of the participants highlighted the presence and support of birth companions of their choice during labour and childbirth. The presence of supportive companions, such as family members and traditional birth attendants, played a key role in shaping the labour and childbirth experiences of adolescents through the provision of emotional and practical support:

*“My neighbour who is a traditional birth attendant went with me and was present in the labour suite during childbirth.”* (Susan, 18-year-old, Para 1)

“*They were helping me [mother-in-law and neighbour] to wash clothes, cook, boil tea and drinking water.”* (Emily, 17-year-old, Para 2)

*“My mother-in-law and even Olwede [pseudonym for the husband] went along with us [transported her to the hospital]. She was allowed [inside the labour suite]. It was Olwede [pseudonym for the husband] that was not in [the labour suite] because he seemed to be having some fear.”* (Sophia, 17-year-old, Para 1)

##### Sub-theme 3.5: Timely care

Participants noted that they received timely care during their childbirth experiences.

*“Immediately I reached, they took me to the examination room, I was examined and told I was about to give birth.”* (Susan, 18-year-old, Para 1)

*“She examined me immediately without wasting time.”* (Sophia, 17-year-old, Para 1)

“I went straight to the nurse and examined me and said the baby was on its way.” (Harper,19-year-old, Para 1)

#### Theme 4: Health facility hygiene

A pervasive theme was identified concerning the pivotal role of hygiene in shaping adolescents’ experiences during childbirth. The nuances of health facility hygiene unfolded through two interconnected dimensions of clean labour suite/ward and clean washrooms (toilets and bathrooms).

##### Sub-theme 4.1: Clean labour suite and ward

Within the health facility, participants consistently appraised the cleanliness of the labour suite and general ward as integral components of their overall experience. Their sentiments, encapsulated in the following excerpts, underscore the significance attributed to the hygiene of these critical spaces:

*“The place was generally clean like the labour suite, the floor was clean because immediately someone delivers the place would be cleaned up and even the toilets.”* (Ella, 16-year-old, Para 1)

*“The hospital [the general ward] was clean and even the labour suite was clean.”* (Grace, 18-year-old, Para 1)

*“Like on the day, I went there were people that had given birth and their attendants had cleaned the labour suite and the general ward even the compound was looking clean.”* (Chloe, 19-year-old, Para 1)

Although each healthcare facility has designated cleaners, in other cases, the adolescents accepted to clean the delivery bed and the labour suite floor themselves to enlist favourable treatment from the healthcare providers as was the case for Chloe:

*“As a person who has just given birth you need to clean the labour bed and floor which I didn’t get any problem with it and that made them [midwives] to like me.”* (Chloe, 19-years-old, Para 1)

##### Sub-theme 4.2: Clean toilet and bathroom

Complementary to the appraisal of the labour suite and ward as clean, participants articulated their satisfaction with the cleanliness of toilet and bathroom facilities within the health facility. This sub-theme underscores adolescents’ discernment of sanitation beyond the immediate context of childbirth, extending to the ancillary areas crucial for their overall well-being. Although it was not very clean, the participants felt that the hygiene of the toilets and the bathrooms was good as cited by some of these participants:

*“The washrooms were generally clean like the bathroom and toilet I had to clean after I had used.”* (Emily, 17-year-old, Para 2)

*“The place was generally clean […] even the toilets, bathrooms.”* (Ella, 16-year-old, Para 1)

*“The bathrooms and toilets were being used well and were clean.”* (Grace, 18-year-old, Para 1)

In other health facilities, either the toilet or bathroom was not clean at any given point in time as noted by some of these participants:

*“The place where I got services from was clean, the toilet was good, it was the bathroom that was not clean and was unbearable.”* (Susan, 18-year-old, Para 1)

### Negative experiences of facility-based childbirth

Although most adolescents experienced positive birth experiences, some of them experienced negative experiences during their care. The negative experiences were in the form of disrespect and abuse and poor amenities and services.

#### Theme 5: Disrespect and abuse

The disrespect and abuse of adolescents during childbirth manifested in six multifaceted dimensions (a) physical abuse, (b) verbal abuse, (c) neglect and abandonment, (d) non-consented care, (e) disempowerment, and (f) bribery and extortion.

##### Sub-theme 5.1: Physical abuse

Physical abuse was one form of D&A experienced by adolescent mothers during childbirth. Some adolescents recounted incidents of physical abuse which included slapping, inappropriate examination, and repair of the episiotomy without anesthesia as noted below:

*“I was stitched [after episiotomy] but it was very painful, I was stitched without put on anesthesia”.* (Lily, 17-year-old Para 1)

One participant described a midwife’s act that resulted in immediate physical harm:

*“Hmm, she [the midwife] gave me a very terrible slap on my thigh, and my thigh got paralysed instantly.”* (Mia, 18-year-old, Para 1)

The presence of relatives of the adolescents did not stop the midwife from physically assaulting the labouring adolescent as noted by one of the participants:

*“I was slapped like twice even and my stepmother was just there looking. [Besides], she was touching [palpating] me in a painful way.”* (Lily, 17-year-old Para 1)

##### Sub-theme 5.2: Verbal abuse

Verbal abuse by midwives was a common form of D&A experienced by these participants during childbirth. They reported that midwives used harsh language and demeaning comments towards them, while others were judgmental and critical of them. Verbal abuse also manifested in the form of threatening adolescents regarding the likely adverse birth outcomes associated with adolescent pregnancies which created a sense of fear among adolescents.

*“They said that I should open up my vagina well because they are not the ones who inserted my husband’s penis into my vagina.”* (Mia, 18-year-old, Para 1)

*“Aaaa, she wasn’t treating me well because the abusive languages she was using were heavy [participant felt shy to verbalise the vulgar words used towards her].”* (Campbell, 19-year-old, Para 2)

*“They then told me that if I push so hard and my uterus gets torn it would be my own problem.”* (Mia, 18-year-old, Para 1)

Some of the adolescents were scolded for being out of school and getting pregnant at a tender age, something they resented and made them feel unwelcome at the health facility.

*“Mm, I was also told I should go back to school that I am still young to be in marriage [started to look away in anger].”* (Emily, 17-year-old, Para 2)

*“I am not the one who told you to get pregnant while you are still young [facial grimace instantly appeared as she recounted her experience].”* (Harper,19-year-old, Para 1)

##### Sub-theme 5.3: Neglect and abandonment

Participants shared distressing accounts of being left alone during labour, such as one of the participants who delivered without assistance. This neglect and abandonment during childbirth exposed adolescents to heightened vulnerability.

*“I delivered alone and the nurse [midwife] only received the baby.”* (Emily, 17-year-old, Para 2)

*“The nurse came when the baby’s head was already out then she came and cut the cord and took the baby on the weigh scale and she moved out because there was no cloth, and my stepmother removed her skirt to cover the baby.”* (Lily, 17-year-old Para 1)

In some instances, the healthcare providers were not present in the hospital and the relatives had to call the healthcare providers from the staff quarters to attend to the adolescent mothers or assist the adolescent mother to give birth without the help of a midwife.

*“By the time I was in labour pain, it was so serious and they went to call the midwife and when she arrived, I had already given birth because my husband didn’t know where the staff room was so he kept knocking one door after the other”.* (Campbell, 19-year-old, Para 2)

*“I started pushing the baby behind the house and I called my mother-in-law, and she found me already pushing then she asked me to go inside to bed. When we reached the bed, the nurse wasn’t around and yet I had the labour urge to push”.* (Mia, 18-year-old, Para 1)

##### Sub-theme 5.4: Non-consented care

Despite positive initial interactions, some of the participants reported that they were not allowed to exercise their rights for self-determination during childbirth which may have violated their rights. Some were denied the opportunity to choose their preferred birthing position while others were examined without seeking their consent.

*“She instructed me to lay and lift my legs which I did but at one point I pushed and then my energy got depleted but she kept on shouting at me that I should hurry up pushing the baby.”* (Grace, 18-year-old, Para 1)

*“They just started the procedure. The Nurse came and inserted her hands in my vagina [without seeking my consent]”* (Sophia, 17-year-old, Para 1)

##### Sub-theme 5.5: Disempowerment

Participants shared experiences of feeling disempowered during healthcare encounters. Disempowerment was perceived by the adolescents as limited opportunities to ask questions regarding their labour process, hospital dynamics, and/or their well-being and that of their babies:

*“I wasn’t given any opportunity to ask even a single question and the way I was treated I could hardly ask any question.”* (Mia, 18-year-old, Para 1)

*“Hmm, I did not ask for any question even I didn’t respond to her [when she was asked].”* (Grace, 18-year-old, Para 1)

In some cases, the midwives covertly placed adolescents under duress to name their babies after them as narrated in the excerpts below:

*“He told me I should name my child after him. I did not tell him [I didn’t want to name my child after him]. I was fearing he would shout at me.”* (Amelia, 18-year-old, Para 2)

##### Sub-theme 5.6: Bribery and extortion

Many of the participants expressed instances of financial exploitation which reflected a system where unofficial payments were often required for various aspects of care. Adolescent mothers felt coerced to comply because of the fear of the likely neglect and abandonment that could result from failure to pay the bribe/extortion fees. In public health facilities in Uganda, mothers are not required to buy any medicine or supplies while receiving care, but this was not the case with the care of some of the adolescents. Some of these unofficial payments were disguised as the mother’s contribution towards buying cleaning agents, gloves, and toilet paper as noted below:

*“She was asking for detergent, washing soap, and then my mother-in-law bought and I said since she has helped me so much, I will get something and visit her at her home.”* (Chloe, 19-year-old, Para 1)

*“In that first examination that she did, there was only one pair of gloves that she used, so when she came back for the second examination there were no gloves in the maama kit that we had and she asked us to pay a sum of Ugx*.[Ugandan Schillings] *2,500* [USD 0.7] *for the gloves, we told her that we had no money.”* (Mia, 18-year-old, Para 1)

In some instances, the bribery and extortion were concealed as payments for birth registration, and birth registration cards were retained until adolescents paid for the card as remarked by some of these participants:

*“We had carried Ugx. 25,000 (USD 7) of which my husband had just borrowed. Everyone was paying for the card and if you have not paid you would not be given the card. Both young and old women were paying the same amount before getting the card.”* (Ella, 16-year-old, Para 1)

*“[…] they again asked for Ugx. 3,000 (USD 0.8) [for the birth registration card] and they said if that money is not there then we would not be discharged when we gave that Ugx. 3,000* [USD 0.8]*, we were discharged.”* (Campbell, 19-year-old, Para 2)

Meanwhile, in other cases, the healthcare providers bluntly asked the mothers to give them money as a token of appreciation, a form of corruption as noted below:

*“[*…*] They asked for some money and I think they were given Ugx. 15.000* [USD 4.2] *because I didn’t pay much attention to that since my mother-in-law was there to handle.”* (Ella, 16-year-old, Para 1)

*“We pleaded saying there is no money [but the midwife insisted on the money] and we gave Ugx. 10,000* [USD 2.8].” (Mia, 18-year-old, Para 1)

#### Theme 6: Health facility constraints

The health facility constraints were critical aspects that shaped the overall experience of adolescents during childbirth. This theme comprises sub-themes that highlight the state of toilets and bathrooms, as well as the stability of the water supply.

##### Sub-theme 6.1: Dirty toilets and bathrooms

Some participants provided varied perspectives on the cleanliness of the facility, specifically highlighting concerns about the state of toilets and bathrooms:

*“It is the toilets that sometimes could be dirty in that you find faeces and urine on the floor and no one bothers to clean it, and the bathrooms sometimes were very dirty. You could find that those women who have just delivered have bathed and their blood flooded on the floor.”* (Chloe, 19-year-old, Para 1)

*“There was so much dirtiness sometimes if you go to bath you find blood on the floor they have not cleaned and then the toilets you find cotton dumped and other dirty things of which if you walk in barefooted you can contract incurable diseases.”* (Lily, 17-year-old Para 1)

Participants expressed that while the hygiene in the labour suite was maintained in some cases, the hygiene in the toilets and bathrooms was poor. This compelled some of them to clean the bathrooms and toilets themselves before use or to simply use the facilities the way they were:

*“The toilets and bathrooms were not clean at all, I had no choice but to [clean it myself] and bath in it like that.”* (Campbell, 19-year-old, Para 2)

*“Where I delivered from was clean […]. You know once there are many people they keep the place dirty, a person urinates or defecates anyhow [….].”* (Grace, 18-year-old, Para 1)

*“The hospital was clean [ward and labour suite] but the toilet was not okay. Inside was very dirty with very bad smell, the floor had a lot of very dirty stuff, and the bathroom had visible human faeces making it very difficult for women like us who had just given birth to bathe from inside.”* (Mia, 18-year-old, Para 1)

##### Sub-theme 6.2: Unstable water supply

Concerns were raised regarding the stability of water supply as a basic amenity required for the maintenance of facility hygiene as well hygiene of clients. Unstable water supply was cited as the main cause of the dirty bathrooms and toilets. These concerns are personified in quotes from some of these adolescents:

*“Sometimes there would be no water most especially if the borehole breaks down and at times when there is water you find that attendants are not willing to do the cleaning.”* (Chloe, 19-years-old, Para 1)

Sometimes, the participants were forced to fetch water from outside the hospital premises due to inconsistent or complete lack of water supply within the facility, which not only added to their physical burden during a vulnerable time but also compromised their access to basic hygiene and sanitation, thereby affecting their overall childbirth experience.

*“There is water, tap water but there was a time when it was off the entire night but then it got back the following morning. Water was being collected from [a secondary school located about 2 km from the health facility] and was far.”* (Susan, 18-year-old, Para 1)

*“There is water, we would collect water from the other side of the hospital [outside the hospital].”* (Mia, 18-year-old, Para 1)

##### Sub-theme 6.3: Shortage of essential medicines and supplies

Some participants shared experiences where there were no essential medicines for managing common complications during pregnancy and childbirth. One of the interviewees never received any treatment during labour because of a lack of medicines although the doctor had told her that she needed medications.

*“I was not injected till I returned home. I was not given any kind of medication and they told me that if I were to be treated then I needed to buy medication which I didn’t have money.”* (Chloe, 19-year-old, Para 1)

For some participants, they were required to purchase all the gloves used during their care. This was after being informed by the healthcare providers that there were no gloves available in the hospital stock.

*“All the gloves that were used are the ones we bought, we bought four pairs”.* (Mia, 18-year-old, Para 1)

## Discussion

This study explored adolescents lived experiences of facility-based childbirth in public health facilities in Northern Uganda. Both positive and negative experiences were reported from most participants. The themes identified in this study align with the experience dimensions of quality of maternal and neonatal care outlined in the WHO Framework for Quality of Maternal and Newborn Care.^45,46^

Previous studies have mostly highlighted the negative experiences of mistreatment of women during childbirth.^12,14,18,47^ Contrary to previous studies, our study noted that adolescents generally reported positive experiences during childbirth ranging from effective communication, dignity and respect, to supportive care, and hygienic health facilities. The positive childbirth experiences could be because they gave birth in lower-level health facilities (H/C III and H/C IV) with less workload, which facilitates individualised care.^48,49^ The adolescent women’s perspectives may also align with Bourdieu’s concept of *‘habitus’* and Shim’s concept of cultural health capital which suggest that vulnerable groups such as adolescents may have lower expectations of healthcare due to their disadvantaged societal position hence reporting more positive childbirth experiences.^50,51^ These findings suggest that healthcare providers need to be cognizant of the diverse experiences and expectations of different demographic groups, such as adolescents, during childbirth, and to tailor their care accordingly to promote positive outcomes and satisfaction with care.

Despite reports of positive experiences, the negative experiences are quite serious, and one could argue that they tip the balance of experiences of these adolescent participants. The forms of mistreatment reported by the study participants included physical and verbal abuse, neglect and abandonment, non-consented care, disempowerment, bribery, and extortion. Participants also reported negative experiences related to dirty toilets and bathrooms, an unstable water supply, and a shortage of essential medicines and supplies. Adolescent participants attributed these negative experiences to lack of birthing necessities, younger age, and perceived low social status by skilled care providers. The poor amenities and provision of services could also be attributed to poor infrastructure maintenance and inefficient supply chain management, all of which lead to substandard conditions.^52–55^ Essential supplies are not only clinical necessities but directly impact perceived respect and dignity and poor infrastructure and supply shortages contribute to systemic disrespect and indirectly discourage future facility-based births. These negative experiences by adolescents are a violation of human rights, of professional practice and exploitation of people at a point of great vulnerability and they contravene multiple international human rights frameworks, including the Respectful Maternity Care Charter, Universal Declaration of Human Rights and the International Convention on Economic, Social and Cultural Rights, which guarantee the right to health, informed consent, and freedom from torture and degrading treatment.^56–58^

These findings align with the results of previous studies conducted in South Africa, Ghana, Guinea, Myanmar, and Nigeria among adolescents, which reported similar accounts of mistreatment during childbirth among adolescents.^12,14,47^ They are also consistent with results of studies conducted in other low-middle-income countries, where poor health facility environment was identified as one of the factors contributing to negative birth experiences.^59–61^ Based on these findings, the authors call for respectful and dignified care during childbirth to ensure a positive childbirth experience.

### Limitations

The results of this study may not be transferrable to adolescents who give birth in private health facilities and urban settings. However, undertaking this study in public health facilities in the rural parts of a low-income country among participants of low socioeconomic status generated new findings in a unique context. These findings could inspire future research on adolescents giving birth in private and urban settings in other contexts to corroborate or challenge the results. This study brought valuable insights into the issues surrounding maternity care, which could have influenced the interpretation of adolescents’ experiences given the background of the authors. Nevertheless, the researchers remained reflexive and aware of their positionality by maintaining a journal throughout the data collection and analysis process. Participants may have been reluctant to fully disclose negative childbirth experiences due to fears of mistreatment or denial of services in future facility visits. To mitigate this, interviews were conducted by someone not directly involved in their care. The interviews were also in participants’ homes to encourage open dialogue. Participants were also reassured that their confidentiality would be preserved throughout the study.

## Conclusions

Adolescents’ lived experiences of facility-based childbirth were multifaceted, encompassing a continuum of positive and negative dimensions. The positive experiences highlighted effective communication, dignity and respect, supportive care, and health facility hygiene, while negative experiences centred around disrespect and abuse, as well as health facility constraints. Emphasising adolescent-friendly childbirth may promote respectful and appropriate care for adolescents. Moreover, the development and implementation of community-based interventions could enhance adolescents’ cultural health capital, enabling them to navigate institutional fields more effectively, recognize and assert their symbolic value, and reposition themselves as legitimate claimants of respectful, adolescent-friendly maternity care.

## Supplemental material

Supplemental material - Adolescents’ lived experiences of facility-based childbirth in rural northern Uganda: A qualitative study Supplemental material for Adolescents’ lived experiences of facility-based childbirth in rural northern Uganda: A qualitative study by Samson Udho and Sheila Elizabeth Clow in Women's Health.

Supplemental material - Adolescents’ lived experiences of facility-based childbirth in rural northern Uganda: A qualitative study Supplemental material for Adolescents’ lived experiences of facility-based childbirth in rural northern Uganda: A qualitative study by Samson Udho and Sheila Elizabeth Clow in Women's Health.

Supplemental material - Adolescents’ lived experiences of facility-based childbirth in rural northern Uganda: A qualitative study Supplemental material for Adolescents’ lived experiences of facility-based childbirth in rural northern Uganda: A qualitative study by Samson Udho and Sheila Elizabeth Clow in Women's Health.

## Data Availability

All relevant data are within the article and its supporting information files. Data is available upon reasonable request from the first author.*
