# Supplementary material for: Adolescents’ lived experiences of facility-based childbirth in rural northern Uganda: A qualitative study
Source: Womens Health (Lond). 2026 Jul 9;22:17455057261468279. doi: 10.1177/17455057261468279 (PMC13351225; doi:10.1177/17455057261468279)
Supplement: Supplemental material - Adolescents’ lived experiences of facility-based childbirth in rural northern Uganda: A qualitative study [file sj-pdf-2-whe-10.1177_17455057261468279.pdf]

Appendix 2: Qualitative In-depth Interview Guide

1.	Study title	Adolescents' Mixed Experiences During Facility-Based Childbirth in Rural Northern Uganda: A Qualitative Study
2.	Introduction	My name is Samson Udho and I am a Doctoral (Ph.D.) student at the University of Cape Town (UCT), South Africa. As part of the fulfillment of my Degree, I am conducting research on what young women, aged 10 to 19 years go through while giving birth in public maternity facilities in Uganda.<br><br>You were selected to participate in the second part of the study because of your unique experiences and ability to express yourself. During our conversation, I will ask you open questions and you'll narrate to me your answer. In case you want to stop talking during our conversation because you're upset and want to collect yourself before continuing, feel free to do so. All the information you share with me will be anonymous & confidential.
3.	Respondent ID	
4.	Date of the interview	
5.	Interview strategies	Repetition, summarising, uh huh, anything else, can you tell me more
6.	Socio-demographic characteristics	1) Could you please tell me more about yourself?
7.	Obstetric history	2) Could you please share with me your history of pregnancy and giving birth?
8.	Disrespect and abuse	3) When you gave birth to your last child, what was that experience like?
9.	Drivers of disrespect and abuse	4) Do you feel you were treated differently from other women who were also giving birth at the same health facility?<br>5) Could you please share with me why do you think you were treated differently?
10.	Closure	I think we are coming to the end of this conversation, is there anything more you would like to add?<br><br>You shared A, B, and C regarding your experiences as an adolescent during childbirth and why you are treated differently. Is this a fair representation of what we talked about?<br><br>Thank you for your time and participation.
