# Supplementary material for: Adolescents’ lived experiences of facility-based childbirth in rural northern Uganda: A qualitative study
Source: Womens Health (Lond). 2026 Jul 9;22:17455057261468279. doi: 10.1177/17455057261468279 (PMC13351225; doi:10.1177/17455057261468279)
Supplement: Supplemental material - Adolescents’ lived experiences of facility-based childbirth in rural northern Uganda: A qualitative study [file sj-pdf-3-whe-10.1177_17455057261468279.pdf]

Appendix 3: Thematic Map of Adolescents' Experiences of Facility-Based Childbirth

CODES/EXTRACTS	SUB-THEME	THEME	OVERARCHING THEME
She told me nothing that would make me have problems with her, IDI 02	**Effective Interaction**	**Effective Communication**	**Adolescents' Mixed Experiences During Facility-Childbirth**
We were even interacting well with joy, IDI 12			
Our conversation was going on well, IDI 12			
She was humble while talking to me and was not rude, IDI 13			
They were talking calmly to me, IDI 13			
She was interacting well with me, IDI 11			
They kept telling me even the time I was giving birth they told me that I should push the baby, IDI 11	**Provision of Information**		
said progress is still long, IDI 13			
I was told there was progress and I was asked to be walking around, IDI 03			
Hum, even how they treated me showed they were respecting me, IDI 11	**Respectful Care**	**Dignity and Respect**	
There was maximum respect, IDI 12			
I was not slapped, IDI 11			
They never shouted at me and they also never slapped me, IDI 13			
They were treating me well, IDI 14			
She started being friendly to me during my pregnancy, IDI 02	**Friendly Care**		
They showed me love, IDI 12			
They treated me very well up to the time that I gave birth, IDI 02			
she loved me at a higher level and wished me a safe delivery, IDI 05			
I would see and tell those people were liking me, IDI 12			
I was given a warm welcome, P014, IDI 01	**Hospitality**	**Supportive Care During Childbirth**	
I was welcomed well, IDI 14, IDI 12			
When I reached the nurse welcomed me, IDI 02			
she received me so well, IDI 05			
They received me without shouting at me, IDI 15			
I was examined and told I was about to give birth, IDI 14	**Encouragement**		
She said I should persevere I will give birth that it's a boy, IDI 11			
The nurse told me that my childbirth would be okay without any complications and also said that young girls are giving birth without complications compared to old women, IDI 05			
She also encouraged me to exercise by helping me to walk around, IDI 02			

"One important thing that I know is the nurse [midwife] was on my side. She delivered me well and made my childbirth very easy without any problem, IDI 05	**Partnership with Adolescents**		
The nurse was there [during labour and childbirth] and she waited to receive the baby, IDI 12			
The nurse was with me up to the time I gave birth. She even cleaned me up, IDI 13			
On getting inside [labour suite], I went straight on bed and both of them [midwives] were inside and I gave birth straight away, IDI 06			
At some point when I would feel like falling down the nurse [midwife] would support me, IDI 02			
My neighbor who is a traditional birth attendant went with me and was present in the labour suite, IDI 14	**Companionship During Labour and Childbirth**		
When I was pushing the baby, there were three people, the nurse [midwife], my sister-in-law, and my mother-in-law, IDI 12			
They were helping me [mother-in-law and neighbor] to wash clothes, cook, boil tea, and drink water, IDI 01			
My mother-in-law and even Jasper [husband] went along with us [transported her to hospital], IDI 05			
She was allowed [inside the labour suite]. It was my husband who was not in [the labour suite] because he seemed to be having some fear, IDI 05			
Immediately I reached, they took me to the examination room, I was examined and told I was about to give birth, IDI 14	**Timely Care**		
She examined me immediately without wasting time, IDI 05			
I went straight to the nurse who examined me and said the baby was on its way, IDI 06			
The labour suit and the general ward even the compound looking clean, IDI 02	**Clean Labour Suite and Ward**	**Health Facility Hygiene**	
The facility was clean even the beds were clean, IDI 08			
Inside the ward was clean, IDI 09			
The place where I got services from was clean, IDI 14			
Where I delivered from was clean, IDI 11			
The hospital was clean and even the labour suite was clean, IDI 03			
The labour suite didn't have any issues, it was clean, IDI 05			
The place was generally clean like the labour suite, the floor was clean because immediately			

someone delivered the place would be cleaned up, and even the toilets, IDI 12			
The washrooms were generally clean like the bathroom and toilet, P001	**Clean Toilets and Bathroom**		
The place was generally clean […] even the toilets, bathrooms, IDI 12			
… but bathrooms were okay, IDI 05			
The bathrooms were being used well and was clean, IDI 03			
The bathroom was okay, IDI 13			
The toilet was clean as they had cleaned it, IDI 11			
The toilet was good, IDI 14			
Hmmmm, she gave me a very terrible slap on my thigh and my thigh got paralysed instantly, IDI 07	**Physical Abuse**	**Disrespect and Abuse**	
I was slapped like twice even and my step mother was just there looking, IDI 04			
….and she was painfully touching me, IDI 04			
They then told me that if I pushed so hard and my uterus got torn it would be my own problem, IDI 07	**Verbal Abuse**		
They said that I should open up my vagina well because they are not the ones who inserted my husband's penis into my vagina, IDI 07			
Mm, I was also told I should go back to school that I am still young to be in marriage, IDI 01			
Aaaa, she wasn't treating me well because the abusive languages she was using were heavy, IDI 10			
I am not the one who told you get pregnant while you are still young, IDI 06			
Mm, the midwife quarreled at me, IDI 04			
… [midwife] put the baby on the weighing scale and left, IDI 04	**Neglect and Abandonment**		
The nurse [midwife] came when the baby's head was already out then she came and cut the cord, IDI 04			
I started pushing the baby behind the house [within the health facility, IDI 07			
I delivered alone and the nurse received the baby, IDI 01			
She left me to push the baby alone as she was busy working at the table while telling me I should push the baby she waited until after I delivered and she even took too long to clean up the blood, IDI 03			
The labour suit was not yet open so I delivered at the door, IDI 10			
The nurse said I can decide to leave you and then she walked away saying that is not how			

people should go the hospital, IDI 04			
By the time I reached [for childbirth], the nurse [midwife] wasn't around and other people opened the door for us, IDI 07			
She instructed me to lay and lift up my legs of which I did but at one point I pushed and then my energy got depleted, IDI 07	**Non-Consented Care**		
They just started the procedure, nurse [midwife] came and inserted her hands in you, IDI 05			
Nurse [midwife] came and inserted her hands in my vagina [without seeking my consent], IDI 05			
I wasn't given any opportunity to ask even a single question and the way I was treated I could hardly ask any question, IDI 07	**Disempowerment**		
Mm, but I did not ask for anything even I didn't respond to her, IDI 03			
I just listened as she talked, IDI 01			
He told me I should name my child after him, IDI 11			
I did not tell her. I was fearing she would shout at me, IDI 05			
What they do is they detain your book (discharge forms) and they are given back after delivering the said items or money, IDI 07	**Bribery and Extortion**		
Everyone was paying for the card [discharge form] and if you had not paid you would not be given the card, IDI 12			
When we were leaving the hospital, we were told to pay Ugx. 13,0000 (USD 3.6). We pleaded saying there was no money and we gave Ugx. 10,000 (USD 2.70), IDI 07			
My husband gave me some money. They asked for Ugx. 3,000 (USD 0.8) and they said if that money was not there then we would not be discharged when we gave that Ugx. 3000 (USD 1), we were discharged, IDI 10			
They asked for some money and I think they were given Ugx. 15.000 (USD 4.2), IDI 12			
Hmm, they just give back your discharge form [after you have paid some money] and then you go and register your child and then get vaccination after which you are free to get back, IDI 07			
Everyone was paying for the card [discharge form] and if you have not paid you would not be given the card, IDI 12			
All the gloves that were used are the ones we bought, we bought four pairs, IDI 07			
I gave them a bucket of washing power and two rolls of toilet papers [as requested], IDI 14			

She was asking for detergent, washing soap and then my mother in-law bought, IDI 02			
They also asked for detergent but we had bought it and this old woman who was taking care of me picked from the paper bag and gave it to them, IDI 10			
The toilets and bathrooms were not clean at all, I had no choice but to bath in it like that, IDI 10	**Dirty Toilets and Bathrooms**	**Health Facility Constraints**	
Inside the ward was clean but the toilets and bathrooms were all not cleaned, IDI 09			
The hospital was clean but the toilet was not okay, inside was very dirty with very bad smell, the floor had a lot of very dirty stuffs, the bathroom had visible human feces making it very difficult for women like us who had just given birth to bathe from inside, IDI 07			
It is the toilets that sometimes could be dirty in that you find feces and urine on the floor and no one bothers to clean it and the bathrooms sometimes were very dirty, you could find that those women who have just delivered have bathed and their blood flooded on the floor, IDI 02			
Sometimes if you go to bath you find blood on the floor they have not cleaned and then the toilets you find cotton dumped and other dirty things, IDI 04			
"In the toilets some people would dump blood out not in the pit which was not good. It is only the toilets that women would dump blood on the floor though some would throw in the pit, IDI 03			
It was the bathroom that was not clean and was unbearable. Some people would go in there and dump in cotton wool soaked in blood, they wouldn't throw it in the pit, IDI 14			
"It was the toilets that were dirty, IDI 05			
It was never clean [toilet and bathroom], ever since I started going for antenatal, there was no single day that I found it clean, IDI 02			
Water wasn't just put off but there seemed to be a problem with the line. Water was being collected from Amach complex and was far, IDI 14	**Unstable Water Supply**		
Sometimes there would be no water most especially if the borehole breaks down, IDI 02			
There is water, we would collect water from the other side of the hospital [outside the hospital] where people are detained, IDI 07			
